# Technique of Peritoneal Catheter Placement under Fluroscopic Guidance

**DOI:** 10.1155/2011/141707

**Published:** 2011-03-30

**Authors:** Ahmed Kamel Abdel-Aal, Santhosh Gaddikeri, Souheil Saddekni

**Affiliations:** Department of Radiology, University of Alabama at Birmingham, 619 19th Street, South Birmingham, AL 35249, USA

## Abstract

Peritoneal catheters are mainly used for peritoneal dialysis in patients with end-stage renal disease. Other uses of this catheter include intraperitoneal chemotherapy and gene therapy for ovarian cancer and draining of uncontrolled refractory ascites in patients with liver cirrhosis. Traditionally, surgeons place most of these peritoneal catheters either by laparoscopy or open laparotomy. We detail our percutaneous approach to placing peritoneal catheters using fluoroscopic guidance. We emphasize the use of additional ultrasound guidance, including gray scale and color Doppler ultrasound, to determine the safest puncture site and to guide the initial needle puncture in order to avoid bowel perforation and injury to epigastric artery. We present our experience in placing peritoneal catheters using this technique in 95 patients with various indications. Fluoroscopic guided percutaneous placement of peritoneal catheters is a safe, minimally invasive, and effective alternative to open surgical or laparoscopic placement.

## 1. Introduction

Peritoneal catheters are mainly used for peritoneal dialysis in patients with end stage renal disease. Other uses of this catheter include intraperitoneal chemotherapy and gene therapy for ovarian cancer and draining of uncontrolled refractory ascites in patients with liver cirrhosis. Peritoneal catheters can be placed either blindly by Seldinger technique or by surgical means (open surgery or laparoscopy). In this paper, we are emphasizing on placement of peritoneal catheter using fluoroscopic guidance [1–4].

Fluoroscopic guided placement of peritoneal catheter is a minimally invasive procedure, and the procedure can be done on an outpatient basis. Very few preprocedure tests (coagulation profile) are required like complete blood count, prothrombin time, and partial thromboplastin time. No bowel preparation is required before the procedure. It can be performed under conscious sedation and thus avoiding the potential complications associated with general anesthesia. In this paper, we will discuss the technique and our experience in peritoneal catheter placement under fluoroscopic guidance [5].

## 2. Technique

Informed signed consent for the interventional procedure is obtained from the patient after explaining the benefits, risks, and alternatives of the procedure. Preprocedure 1 gm of IV cefazoline (or 500 mg of vancomycin if the patient is allergic to cefazoline) is given 1 hour prior to the procedure. The patient is placed supine on the procedure table and his abdomen is prepared and draped in aseptic fashion. The procedure is usually performed under conscious sedation using intravenous midazolam hydrochloride and fentanyl citrate. Patient was continuously monitored for his vitals, ECG, and oxygen saturation throughout the procedure. The conscious sedation was repeated when required during the procedure.

The chances of bowel perforation and inferior epigastric artery injury are considerably less when ultrasound guidance is used for initial placement of needle in the peritoneal cavity. Real-time ultrasonography is performed first to determine a safe puncture site where there is maximum separation between the bowel and anterior abdominal wall to minimize the risk of bowel perforation. Ultrasound scanning is commenced at the paraumbilical region (either left or right) approximately 2 cm inferior and lateral to the umbilicus. On ultrasonography, the subcutaneous tissue is seen as superficial hypoechoic band-like structure and, the rectus abdominis muscle is a deeper hypoechoic band with multiple linear high specular echoes. The parietal peritoneum is seen as thin echogenic line just deep to the rectus abdominis muscle. Bowel loops containing gas will produce a ring-down artifact and posterior shadowing. Since the cuff of the peritoneal catheter should be within the rectus muscle, we prefer to puncture through the thickest mid portion of the rectus muscle. We always try to avoid the lateral margin of the rectus because chances of dislodgement of cuff either within the abdominal cavity or in subcutaneous tissue are very high. Color Doppler is also performed in the same setting to avoid penetrating and transecting the inferior epigastric artery that might lead to intramuscular hematoma. 

**Figure 1:**
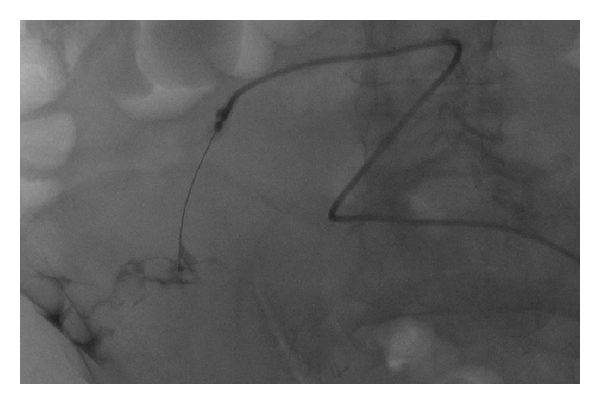
Fluoroscopic image obtained after placement of micropuncture needle in the peritoneal cavity, showing small amount of nonionic contrast material outlining the bowel wall and confirming the intraperitoneal location of the needle.

**Figure 2:**
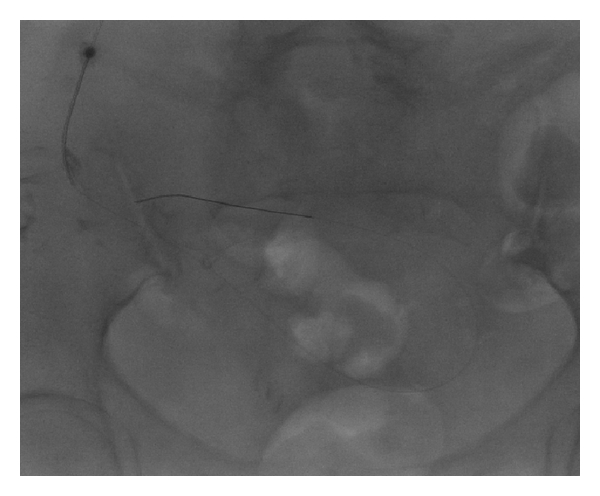
Fluoroscopic image obtained after insertion of cope wire through the needle into the peritoneal cavity.

**Figure 3:**
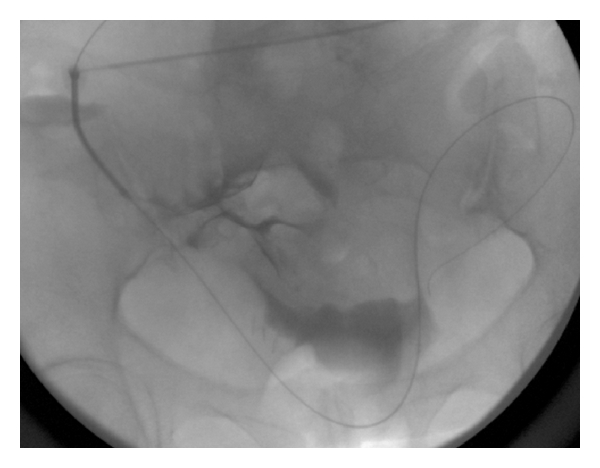
Fluoroscopic image obtained after placement of 6-French sheath. A stiff glide wire is passed through the sheath into the peritoneal cavity. Note the small amount of contrast material in the pelvic peritoneal cavity.

Once the safe site is selected for puncture, 1% lidocaine is than applied in the anticipated puncture site for local anesthesia. Under ultrasound guidance, a 21 gauge micropuncture needle (Stiffen microintroducer kit, Galt medical group) is inserted with a 45 degree caudal angulation through the anesthetized region. Since the parietal peritoneum is sensitive to pain, its transgression will cause some discomfort to the patient. A small amount of nonionic contrast is injected through the needle to confirm its location within the peritoneal cavity (Figure 1). A 0.018 inch cope wire that comes with the microintroducer kit is then advanced into the peritoneal cavity through the needle (Figure 2). The needle is then exchanged over the wire for a 4-French microsheath that also comes with the microintroducer kit. A small amount of nonionic contrast is again injected through the sheath to confirm its location in the peritoneal cavity. If patient has ascites, the position can be confirmed by aspirating a small amount of ascitic fluid besides injecting the nonionic contrast. The cope wire is than removed, and a 0.035 inch stiff glide wire (glide wire, Terumo, Tokyo, Japan) is advanced into the peritoneal cavity under fluoroscopic guidance towards the pelvis. The 4-French microsheath is than exchanged for a 6-French introducer sheath (Pinnacle, Terumo, Tokyo, Japan) over the stiff glide wire. A small amount of contrast is again injected through the 6-French sheath to confirm that the sheath is located in the peritoneal cavity (Figure 3). 

**Figure 4:**
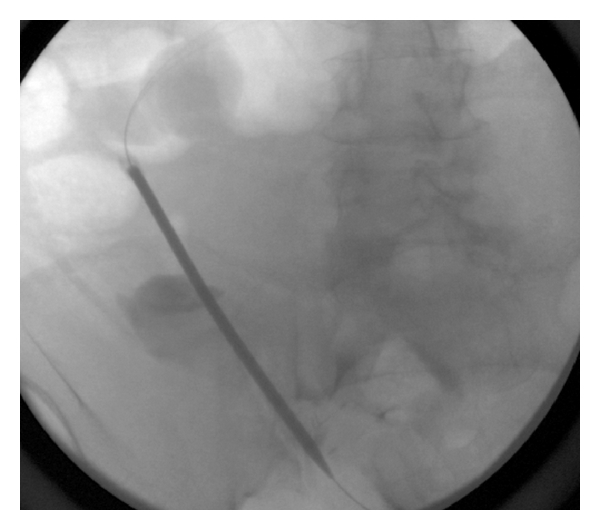
Fluoroscopic image showing 16-French peel-away sheath placed in the peritoneal cavity over the stiff glide wire.

If there is no ascites, 300 cc of normal saline are delivered through the sheath into the peritoneal cavity to displace the bowel loops. Displacing the bowel loops creates a space for the easy placement of the peritoneal catheter into the peritoneal cavity. A 2 cms incision is made at the site of sheath insertion, and blunt dissection is made using a curved hemostat down to the rectus abdominis muscle. Next, the sheath is removed over the wire, and the tract is serially dilated using 8-, 12-, and 14-French dilators (Hydrophilic coated dilator, Cook, Bloomington, IN) over the stiff glide wire. After this, a 16-French, 15 cms peel-away sheath which is included in the peritoneal catheter kit (Quinton Curl Cath, Tyco health care, Mansfield, MA) is introduced into the peritoneal cavity over the glide wire (Figure 4). If the kit does not have the peel-away sheath, then an 18-French, 15 cms (Peel-away introducer set, Cook, Bloomington, IN) can be alternatively used. 

**Figure 5:**
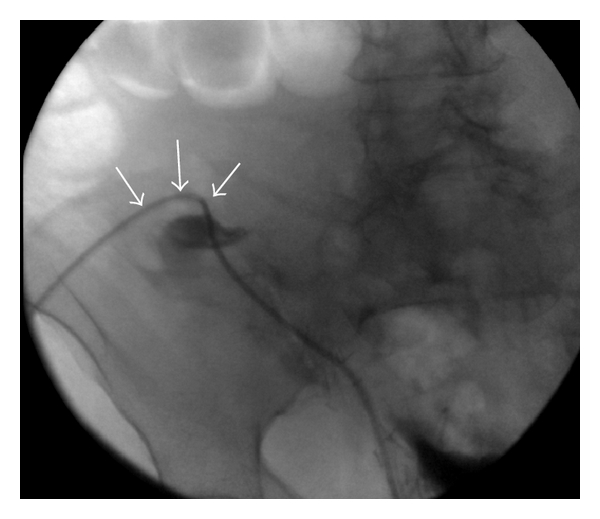
Fluoroscopic image after injection of small amount of contrast material into the catheter showing no evidence of kink in the tunneled portion (arrows).

Next, the anticipated tunneling site, usually 4 cm inferior and lateral to the original incision, is anesthetized with 1% lidocaine. A 5 mm incision is made at the anticipated exit site of the catheter from the tunnel. The outer end of the catheter (noncurled) is mounted on the tunneler/tunneling stylet, and the catheter is tunneled. The outer cuff is buried in the subcutaneous tissue of the tunnel. The inner dilator of the peel-away sheath is then removed, and a 57-cms double-cuffed swan neck peritoneal catheter (Quinton Curl Cath, Tyco health care, Mansfield, MA) is then introduced over the stiff glide wire through the peel-away sheath into the pelvic peritoneal cavity. The peel-away sheath is then removed while exerting an inward pressure on the peritoneal catheter to implant the inner cuff of the peritoneal catheter in the rectus abdominis muscle. The plastic adapter that comes in the peritoneal catheter kit is then connected to the outer end of the catheter. A small amount of contrast is then injected through the catheter under fluoroscopy to exclude any kinks in the catheter and to confirm correct location of its tip in the peritoneal cavity (Figure 5).

The subcutaneous tissue of the first incision site is approximated and sutured using 2-0 Vicryl absorbable sutures, and the skin incision is sutured using 4-0 Monocryl absorbable sutures. An extension catheter (transfer set, Baxter) is connected to the plastic adapter if the catheter was placed for peritoneal dialysis. The outer portion of the catheter, the adapter, and the extension catheter are covered with two pieces of gauze (10 × 10 cm) and then covered by transparent dressing (Tegaderm, 3 M healthcare). The transparent dressing should not be applied directly to the catheter without the gauze, since this will risk catheter dislodgement whenever the dressing is removed.

Postprocedure care needs to be followed to avoid catheter infection. Dressing should be changed after 2 days and then every week by a health care provider. The exit wound should be kept dry till the wound heals. Bathing should be avoided except for sponge baths. Heavy weight lifting (>10 Lbs or 4.5 Kgs) should also avoided for the first four weeks.

## 3. Discussion

Chronic renal failure requiring dialysis remains the most common indication for placement of peritoneal catheters. The major advantage of peritoneal dialysis over hemodialysis is the more patient independence, since peritoneal dialysis allows for more flexible scheduling and can be performed at home. On the other hand, hemodialysis patients are required to adhere to a specific schedule and travel to the dialysis unit. Hemodialysis also requires strict diet control and fluid control more than peritoneal dialysis. However, peritoneal catheters should be placed in a motivated and reliable patient, when peritoneal dialysis is considered the best modality for their treatment [1, 5, 6]. Recently, several new indications evolved for placement of peritoneal catheter including palliation of malignant ascites [5, 7, 8] and for delivery of intraperitoneal chemotherapy [5]. 

Traditionally, surgeons place most of these peritoneal catheters either by laparoscopy or open laparotomy. Lately, interventional radiologists have been placing them using fluoroscopic guidance [5]. 

There are two major perioperative complications of placing peritoneal catheters. First is bowel perforation with subsequent peritonitis. Second is injury of the epigastric artery with a subsequent extensive hematoma in the rectus abdominis muscle. Initial ultrasound guided needle placement allows for identification of the abdominal wall structures and bowel loops thus avoiding bowel wall puncture. On the other hand, color Doppler imaging allows identification of the epigastric vessels thus preventing their injury and subsequent abdominal wall hematoma. Once the needle is in place, the injection of contrast media under fluoroscopy verifies the correct placement of the needle in the peritoneal cavity by outlining the bowel walls and pelvic organs. 

The most common late complication of catheter placement is the leakage of fluid (dialysate or ascites) around the catheter, which has been reported in 8.6% to 24% of cases [9]. Several of the techniques that we employ including the use of a double cuff, the insertion of the inner cuff into the rectus abdominis muscle, and the swan-neck configuration of the peritoneal catheters have been reported to help decrease the incidence of this complication [5, 10]. 

From January 2005 through November 2010, we placed a total of 95 peritoneal catheters using the above technique. Indications included end-stage renal disease requiring dialysis in 58 patients, intraperitoneal gene therapy/chemotherapy for metastatic ovarian carcinoma (as part of an oncology trial protocol) in 32 patients, and refractory ascites requiring frequent large volume paracenthesis in 5 patients. Technical success rate was 97.9%. The complication rate was 2.1% (2 patients). Complications included two bowel punctures, and in both cases, the procedure was terminated. The patients were treated with broad-spectrum intravenous antibiotics and overnight observation, without further complications. Successful placement was achieved in these two patients following a second attempt a week later.

Minor technical challenges were sometimes encountered in patients in the gene therapy/chemotherapy trial group. These challenges were attributed to intraperitoneal adhesions from prior abdominal surgeries related to patients' disease resulting in contrast loculation and difficulty passing the stiff glide wire down to the pelvic cavity. This was usually overcome either by using a different puncture site or by using 4-French Bernstein catheter (Cook, Bloomington, Indiana) inserted through the 6-French sheath to redirect the stiff glide wire in the peritoneal cavity away from adhesions and into the dependant portion of the pelvic cavity.

## 4. Conclusion

There are several indications for placement of peritoneal catheters, with peritoneal dialysis being the most common indication. The technique described in this paper for percutaneous placement of peritoneal catheters is an outpatient procedure performed under conscious sedation. The use of fluoroscopic guidance for placement of peritoneal catheters makes it safer, relatively less expensive, and minimally invasive compared to surgery or laparoscopy.
